# Thromboembolism after cardiac surgery in a patient with cancer-related thrombosis and severe thrombocytopenia: A case report

**DOI:** 10.1097/MD.0000000000026152

**Published:** 2021-06-04

**Authors:** Soo Yeon Kim, Soo Jin Ro, Su Rim Koh, Hyunyoung Lim

**Affiliations:** Department of Anesthesiology and Pain Medicine, Hanyang University Medical Center, Hanyang University College of Medicine, Seoul, Korea.

**Keywords:** cancer-associated thrombosis, cardiac surgery, thrombocytopenia, venous thromboembolism

## Abstract

**Rationale:**

Patients with cancer have elevated risk of both venous thromboembolism and bleeding compared with patients without cancer due to cancer- and patient-specific factors. Balancing the increased and competing risks of clotting and bleeding in these patients can be difficult because management of cancer-associated thrombosis requires anticoagulation despite its known increased risks for bleeding. The adjustment of blood transfusion or cessation of anticoagulants can be a challenge in surgical diagnosis or treatment of cancer patients with such an imbalanced coagulate status.

**Patient concerns:**

A 45-year-old woman with no underlying disease was suspected of ovarian cancer and was awaiting diagnostic laparoscopic exploration surgery.

**Diagnoses:**

While waiting for the surgery, the patient developed chest pain and underwent stent insertion under diagnosis of myocardial infarction. Two weeks later, endocarditis developed, and replacement of the aortic valve and mitral valve was planned. In addition, the patient developed multiple thromboembolisms and was administered anticoagulants to eliminate vegetation of valves and multiple thromboses. Her blood test showed anemia (7.4 g/dL) and severe thrombocytopenia (24 × 10^9^/L).

**Interventions:**

The patient underwent double valve replacement.

**Outcomes:**

A color change of the left lower extremity was noted 5 hours after double valve replacement, and angiography was performed. Thrombectomy was performed under diagnosis of thrombosis in the left iliac artery. One month later, the patient underwent laparoscopic exploration surgery as scheduled.

**Lessons:**

This case will help establish the criteria of blood coagulation for surgical treatment of cancer patients with imbalanced clotting and bleeding.

## 1 Introduction

Cancer is closely related to various types of coagulopathy such as increased risk of venous thromboembolism (VTE).^[1]^ Therefore, antithrombotic medications might be needed in cancer patients. These patients have varying incidence, depth, and duration of thrombocytopenia depending on cancer type, anticancer treatment, bone marrow involvement, and comorbidities. Cancer patients also have an increased risk of bleeding when on antithrombotic medications compared with non-cancer patients.^[2]^ This bleeding risk is increased by the thrombocytopenia common in hematologic malignancies and chemotherapy regimens. Management of cancer-associated thrombosis requires antithrombotic medications despite their known increased risks for bleeding. Therefore, balancing the risks of thrombosis and bleeding in cancer patients can be a challenge.

Platelets play a very important role in postoperative hemostasis, especially after cardiac surgery.^[3]^ Postoperative excessive bleeding can lead to massive transfusion and increase in postoperative morbidity and mortality. In particular, heart surgery using cardiopulmonary bypass (CPB) can lead to heparin-induced thrombocytopenia and thrombosis, so patients with thrombocytopenia (platelet counts <150 × 10^9^/L) can have a high risk of bleeding complications if they undergo heart surgery.

Antithrombotic management is informed mainly by expert opinion and several recent retrospective studies on VTE.^[1]^ Nevertheless, appreciate guidelines for antithrombotic therapy in these patients have not been clearly established. In addition, there have been no reports of heart surgery using CPB in cancer patient with very severe thrombocytopenia (platelet count <30 × 10^9^/L). We report a case of thromboembolism following double valve replacement in a cancer patient with life-threatening thrombocytopenia and recurrent thrombosis.

## 2 Case report

**Figure 1:**
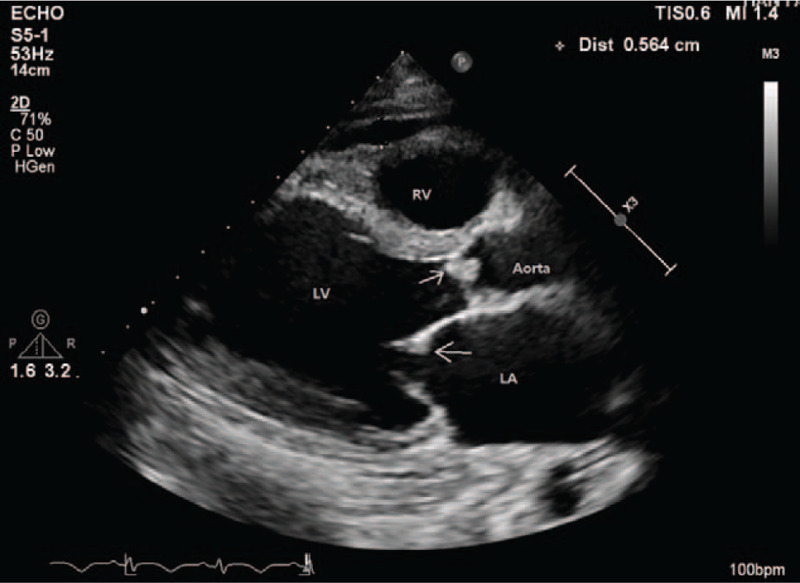
Echocardiographic findings before surgery. In the parasternal long-axis view, the arrow indicates significant vegetations in aortic and mitral valves. LA = left atrium, LV = left ventricle, RV = right ventricle.

A 45-year-old woman with no underlying disease had increasing vaginal bleeding and menorrhagia for 1 year. She visited the hospital due to fatigue and bilateral calf pain, and radiology showed malignancy lesions of the uterus, ovary, spine, and pelvic bone. She was suspected of ovarian cancer and was awaiting diagnostic laparoscopic exploration surgery. While waiting for the surgery, the patient developed chest pain and underwent stent insertion of the left anterior descending artery under diagnosis of myocardial infarction. She was prescribed aspirin and ticagrelor, but increased vaginal bleeding prompted a switch to aspirin and plavix. After 2 weeks, she developed sudden dyspnea and was admitted to the hospital. Abdominal computed tomography showed left ovary cancer, right hepatic vein thrombosis, splenic infarction, and bilateral renal infarction. Peripheral blood cell morphology test showed normocytic and normochromic red blood cells (RBC) and normocytic platelets with a reduced count. Echocardiographic findings showed thrombosis similar to the vegetation of endocarditis in the aortic valve and mitral valve (Fig. 1), and double valve replacement was planned. She was administered antibiotics, and an adjusted dose of low-molecular weight heparin (LMWH) was given to eliminate vegetation of valves and recurrent VTE; however, this treatment was stopped due to anemia (RBC: 6–8 g/dL) and severe thrombocytopenia (platelet counts: 10–24 × 10^9^/L).

The day before surgery, the patient received 1 unit of apheresis platelet (6 pooled platelets following apheresis of donor whole blood) transfusion. After transfusion, the platelet count was improved to 40 × 10^9^/L. On the day of surgery, the platelet count had dropped to 24 × 10^9^/L. The surgery started, and after sternotomy, 150 mg of heparin was injected, and the activated clotting time (ACT) was 526 seconds. After cannulation of the aorta, right atrium, and inferior vena cava, CPB was initiated. The aortic valve and mitral valve were replaced by mechanical valves without complication. The patient was weaned from CPB, and vascular cannulations were removed. Dobutamine, nitroglycerine, and diltiazem were infused to facilitate CPB weaning and maintain blood pressure. Total CPB time was 280 minutes, ACT was 109 seconds after 150 mg of protamine was injected slowly, and total operation time was 420 minutes. Throughout the procedure, the patient received 4250 mL of plasma solution A, 8 units of packed RBC, 16 units of cryoprecipitate, 2 units of apheresis platelet, and 16 units of platelet concentrate. Estimated intraoperative blood loss was 4000 mL, and total urine output was 5300 mL. After surgery, the patient was transferred to the intensive care unit in a stable state. In the postoperative blood sample test, hemoglobin 9.5 g/dL, platelet counts 89 × 10^9^/L, and prothrombin time (PT) international normalized ratio (INR) 1.3 were measured.

A color change of the left lower extremity was found 5 hours after double valve replacement, and angiography showed thrombosis in the left common iliac, femoral, and popliteal arteries. Emergent thrombectomy was performed, and she was weaned from mechanical ventilation the next day. On the first post-operative day, hemoglobin 11.9 g/dL, platelet count 58 × 10^9^/L, and PT INR 1.19 were measured, and warfarin 5 mg and aspirin 100 mg administration was started. One month later, the patient underwent laparoscopic exploration surgery as scheduled. The patient died of worsening disseminated intravascular coagulopathy and heart failure after the surgery. Written informed consent for publication of patient information and images was provided by the legal representatives.

## 3 Discussion

Cancer is associated with increased risk of VTE.^[1]^ Cancer-related VTE challenges clinicians as it can produce increased risk of recurrent VTE, bleeding complications, morbidity, and mortality. In particular, hematologic malignancies are often related to thrombocytopenia secondary to bone marrow infiltration and/or myelotoxic effects of treatments, leading to increased risk of bleeding.^[4]^ Although there are insufficient data available to develop evidence-based guidelines, there have been several studies on reduced administration of anticoagulants such as LMWH to cancer patients with VTE and thrombocytopenia according to platelet count and duration of VTE onset.^[1,2,4]^ Acute VTE is the major risk of developing recurrent rather than chronic status.^[4]^ Therefore, control of appropriate anticoagulant doses is important. A platelet count of 50x10^9^/L is the empirical cut-off level based on the concurrence that a lower level increases the risk of spontaneous bleeding. Some authors suggest using platelet transfusion to increase the platelet count by 50 × 10^9^/L or more while using anticoagulants for safe anticoagulation.^[1,5]^ Napolitano et al suggested that insertion of an inferior vena cava (IVC) filter was appropriate in acute and recurrent VTE in cases of severe thrombocytopenia (platelet counts <30 × 10^9^/L).^[4]^ The patient in this report had acute and multiple VTEs in the hepatic vein and splenic and renal arteries and had consistent severe thrombocytopenia (platelet counts <30 × 10^9^/L). Therefore, the IVC filter could have been a good choice to prevent vegetation in the heart valve.

Preoperative thrombocytopenia could be associated with increased risk of bleeding after surgery. Particularly in heart surgery, CPB can cause excessive bleeding after surgery, leading to increased mortality, morbidity, transfusion requirements, and re-intervention.^[2]^ CPB is associated with platelet and coagulation defects, inflammation, and increased fibrinolysis and exacerbates hemostasis by releasing numerous vasoactive substances and cytotoxic chemicals that affect coagulation, vascular resistance, vascular permeability, fluid balance, and major organ function. Hypothermia, hemodilution, and hypoperfusion during CPB also can be responsible for increased bleeding risk after surgery.^[6]^ In general, heparin is used before cannulation of CPB. In patients undergoing heart surgery, heparin-induced thrombocytopenia (HIT) occurs in 1 to 3% due to formation of antibodies against the heparin and platelet factor 4 antibody complex.^[3,7,8]^ The initial pre-CPB dose of heparin for anticoagulation is recommended at 300 U/kg and prevents fibrin monomer appearance during CPB when the ACT is above 400 seconds. If lower doses of heparin are used, thrombin production can be increased, which can reduce the hemostatic function of platelets, leading to increased bleeding risk after CPB.^[6]^ In this case, 150 mg (15,000 U) of heparin was used before CPB, and ACT of 526 seconds was confirmed after 15 minutes. During CPB, the maximal ACT was 873 and 109 seconds after heparin reversal with protamine. In patients with preoperative severe thrombocytopenia, use of routine doses of heparin, similar to that of common patients, did not affect ACT. However, to provide accurate guidelines, further research is needed on appropriate heparin dose for CPB in patients with severe thrombocytopenia.

Ashoub et al reported a case with a very low platelet count (<20 × 10^9^/L) that demonstrated safe and successful cardiac surgery in patients with thrombocytopenia through appropriate platelet transfusion and coagulation monitoring during surgery.^[2]^ This indicates that, even if the platelet count (<50 × 10^9^/L) is very low, there is no need to postpone heart surgery. Kuhl et al reported a case in which the combination of left ventricular failure, use of extracorporeal circulatory assist, and increased thrombogenic disposition due to HIT caused a fatal course with a recurrent massive ventricular thrombus, hemorrhagic lung edema, and additional septic shock.^[8]^ In that case, the patient was transfused with 1 unit apheresis platelet, 16 units platelet concentrate, and 16 units cryoprecipitate without platelet count or thromboelastogram monitoring after CPB weaning. The VTE in the left lower extremity that can develop after heart surgery might have occurred as a result of HIT, excessive platelet transfusion, or a combination of these. However, it is difficult to determine an appropriate platelet transfusion amount because the result of platelet count or thromboelastography cannot be confirmed quickly enough to allow immediate response.

In conclusion, control of coagulopathy in cancer patients with both severe thrombocytopenia and recurrent VTE requires the use of appropriate criteria for individual patients and close monitoring and careful judgement. Excessive attempts to normalize platelet count and coagulation in cardiac surgery worsen the prognosis of such patient. Further studies are needed to establish guidelines for heparin dose and platelet transfusion for CPB in these patients undergoing heart surgery.

## Author contributions

**Data curation:** Su Rim Koh.

**Supervision:** Hyunyoung Lim.

**Writing – original draft:** Soo Yeon Kim, Soo Jin Ro.
